# Supplementary material for: Psychological safety in European medical students’ last supervised patient encounter: A cross-sectional survey
Source: PLoS One. 2023 Apr 27;18(4):e0285014. doi: 10.1371/journal.pone.0285014 (PMC10138856; doi:10.1371/journal.pone.0285014)
Supplement: S1 File — (DOCX) [file pone.0285014.s001.docx]

# Codebook

The general setup of this codebook is as follows

# Part of survey

## Raw data, i.e. data downloaded directly from online survey

--------------------------------------------------------------------------------------------------------------------------------------------------------------------------------

Variable name

Description of how data were obtained

--------------------------------------------------------------------------------------------------------------------------------------------------------------------------------

Description of variable type and values

--------------------------------------------------------------------------------------------------------------------------------------------------------------------------------

Variable

Description of how data were obtained

--------------------------------------------------------------------------------------------------------------------------------------------------------------------------------

Description of variable type and values

## Transformed data, i.e. data after some form of manipulation of raw data

--------------------------------------------------------------------------------------------------------------------------------------------------------------------------------

Variable name

Description of how data were obtained

--------------------------------------------------------------------------------------------------------------------------------------------------------------------------------

Description of variable type and values

--------------------------------------------------------------------------------------------------------------------------------------------------------------------------------

Variable

Description of how data were obtained

--------------------------------------------------------------------------------------------------------------------------------------------------------------------------------

Description of variable type and values

# Background

## Raw data

--------------------------------------------------------------------------------------------------------------------------------------------------------------------------------

NorwegianStudentAbroud

Based on what link students had used to respond to the survey: Students responding to the survey for Norwegian medical students abroad here have the value “yes”, while those who responded to the survey targeted to European medical students in general have the value “no”

--------------------------------------------------------------------------------------------------------------------------------------------------------------------------------

Type: String (str3)

Unique values: 2 Missing "": 0/908

Tabulation: Freq. Value

682 "No"

226 "Yes"

-------------------------------------------------------------------------------------------------------------------------------------------------------------------------------

Age

Response to question: How old are you (in years)?

--------------------------------------------------------------------------------------------------------------------------------------------------------------------------------

Type: String (str22)

Unique values: 15 Missing "": 21/908

Possible values are 18 to 30 and >30

--------------------------------------------------------------------------------------------------------------------------------------------------------------------------------

Whichgenderdoyouidentifywit

Response to question: Which gender do you identify with?

--------------------------------------------------------------------------------------------------------------------------------------------------------------------------------

Type: String (str22)

Unique values: 4 Missing "": 1/908

Tabulation: Freq. Value

1 ""

3 "Do not wish to specify"

659 "Female"

243 "Male"

2 "Non-binary"

--------------------------------------------------------------------------------------------------------------------------------------------------------------------------------

Inwhichcountrydoyoustudyme

Response to question: In which country do you study medicine?

--------------------------------------------------------------------------------------------------------------------------------------------------------------------------------

Type: String (str26), but longest is str22

Unique values: 26 Missing "": 2/908

Set response. Response options if Norwegian student abroad:

Bjørknes Høyskole

Czechia

Denmark

Hungary

Latvia

Poland

Slovakia

Do not wish to specify

Other European country

Other non-European country

Response options if answering link to European medical students, not Norwegian abroad:

Albania

Andorra

Armenia

Austria

Belarus

Belgium

Bosnia and Herzegovina

Bulgaria

Croatia

Cyprus

Czechia

Denmark

Estonia

Finland

France

Georgia

Germany

Greece

Hungary

Iceland

Italy

Latvia

Lithuania

Luxemburg

Malta

Moldova

Monaco

Montenegro

Netherlands

North Macedonia

Norway

Poland

Portugal

Romania

San Marino

Serbia

Slovakia

Slovenia

Spain

Sweden

Switzerland

Turkey

Ukraine

United Kingdom

Other European country

Africa

Asia

North America

Oceania

South America

Do not wish to specify

--------------------------------------------------------------------------------------------------------------------------------------------------------------------------------

MedSchoolDuration

Response to question: What number of years is your medical school curriculum?

--------------------------------------------------------------------------------------------------------------------------------------------------------------------------------

Type: Numeric (byte)

Range: [2,7] Units: 1

Unique values: 6 Missing .: 10/908

Tabulation: Freq. Value

7 2

8 3

20 4

22 5

807 6

34 7

10 .

--------------------------------------------------------------------------------------------------------------------------------------------------------------------------------

MedSchoolYear

Response to question: Which year of medical school are you in?

--------------------------------------------------------------------------------------------------------------------------------------------------------------------------------

Type: Numeric (byte)

Range: [1,7] Units: 1

Unique values: 7 Missing .: 1/908

Tabulation: Freq. Value

31 1

60 2

152 3

228 4

227 5

206 6

3 7

1 .

--------------------------------------------------------------------------------------------------------------------------------------------------------------------------------

SeePatients

Response to question: In which year of medical school did you start interacting with patients?

--------------------------------------------------------------------------------------------------------------------------------------------------------------------------------

Type: Numeric (byte)

Range: [1,6] Units: 1

Unique values: 6 Missing .: 7/908

Tabulation: Freq. Value

286 1

162 2

390 3

54 4

8 5

1 6

7 .

## Transformed data

--------------------------------------------------------------------------------------------------------------------------------------------------------------------------------

NNNorway

Combination of data on which link students used (NorwegianStudentAbroud) to respond to the survey, i.e. whether they were a Norwegian studying abroad or another European medical student, and country (Inwhichcountrydoyoustudyme) to categorise into three groups: Studying in Norway, non-Norwegian studying outside of Norway or Norwegian studying abroad.

--------------------------------------------------------------------------------------------------------------------------------------------------------------------------------

Type: Numeric (float)

Range: [0,2] Units: 1

Unique values: 3 Missing .: 2/908

Tabulation: Freq. Value Label

451 0 Studying in Norway

229 1 Non-Norwegian studying outside Norway

226 2 Norwegian studying abroad

2 . Missing value

--------------------------------------------------------------------------------------------------------------------------------------------------------------------------------

Age_c

Recoded response to “How old are you (in years)?” from text to numbers

--------------------------------------------------------------------------------------------------------------------------------------------------------------------------------

Type: Numeric (long)

Label: Age_c

Range: [1,15] Units: 1

Unique values: 15 Missing .: 21/908

--------------------------------------------------------------------------------------------------------------------------------------------------------------------------------

Age_6c

Divided responses to “How old are you (in years?)” into 6 groups based on response as described under “Label”

--------------------------------------------------------------------------------------------------------------------------------------------------------------------------------

Type: Numeric (long)

Label: Age_6c

Range: [1,6] Units: 1

Unique values: 6 Missing .: 22/908

Tabulation: Freq. Numeric Label

155 1 less than 22

121 2 22

140 3 23

145 4 24

182 5 25-26

143 6 27 or above

22 . Missing value

--------------------------------------------------------------------------------------------------------------------------------------------------------------------------------

Age_cg

Divided responses to “How old are you (in years?)” into 4 groups based on response as described under “Label”

--------------------------------------------------------------------------------------------------------------------------------------------------------------------------------

Type: Numeric (long)

Label: Age_cg

Range: [1,5] Units: 1

Unique values: 5 Missing .: 21/908

Tabulation: Freq. Numeric Label

155 1 18-21

406 2 22-24

229 3 25-27

96 4 >27

1 5 Do not wish to specify

21 . Missing value

--------------------------------------------------------------------------------------------------------------------------------------------------------------------------------

Gender

Response to question “Which gender do you identify with?” with addition of numeric value

--------------------------------------------------------------------------------------------------------------------------------------------------------------------------------

Type: Numeric (long)

Label: Dummy

Range: [1,4] Units: 1

Unique values: 4 Missing .: 1/908

Tabulation: Freq. Numeric Label

3 1 Do not wish to specify

659 2 Female

243 3 Male

2 4 Non-binary

1 . Missing value

--------------------------------------------------------------------------------------------------------------------------------------------------------------------------------

MaleGender

RECODE of Gender (Response to “Which gender do you identify with?”), where those who responded “male” is assigned 1 and all other responses are assigned 0.

--------------------------------------------------------------------------------------------------------------------------------------------------------------------------------

Type: Numeric (long)

Label: MaleGender

Range: [0,1] Units: 1

Unique values: 2 Missing .: 1/908

Tabulation: Freq. Numeric Label

664 0 NotMale

243 1 Male

1 . Missing value

--------------------------------------------------------------------------------------------------------------------------------------------------------------------------------

Country

Recode of response to “In which country do you study medicine?” so that each country is assigned a number

--------------------------------------------------------------------------------------------------------------------------------------------------------------------------------

Type: Numeric (long)

Label: Country

Range: [3,30] Units: 1

Unique values: 26 Missing .: 2/908

Examples: 9 Denmark

11 Greece

18 Norway

21 Poland

--------------------------------------------------------------------------------------------------------------------------------------------------------------------------------

StudInNorway

RECODE of Country (Response to “In which country do you study medicine?”). So that those who study in Norway are assigned the value 1 and all other responses are assigned 0

--------------------------------------------------------------------------------------------------------------------------------------------------------------------------------

Type: Numeric (long)

Label: StudInNorway

Range: [0,1] Units: 1

Unique values: 2 Missing .: 2/908

Tabulation: Freq. Numeric Label

677 0 Not Norway

229 1 Norway

2 . Missing value

--------------------------------------------------------------------------------------------------------------------------------------------------------------------------------

EuropeanRegion

RECODE of Country (Response to “In which country do you study medicine?”), so that countries are divided into European region based on the UN M49 classification.

--------------------------------------------------------------------------------------------------------------------------------------------------------------------------------

Type: Numeric (long)

Label: EuropeanRegion

Range: [0,4] Units: 1

Unique values: 5 Missing .: 2/908

Tabulation: Freq. Numeric Label

294 0 Northern Europe

273 1 Eastern Europe

107 2 Southern Europe

194 3 Western Europe

38 4 Other European country

2 . Missing value

--------------------------------------------------------------------------------------------------------------------------------------------------------------------------------

ProgressionMedSchool

Respondent's year in medical school divided by medical school duration (Response to question “Which year of medical school are you in?” divided by response to question “What number of years is your medical school curriculum”)

--------------------------------------------------------------------------------------------------------------------------------------------------------------------------------

Type: Numeric (float)

Range: [.16666667,1] Units: 1.000e-08

Unique values: 17 Missing .: 11/908

Mean: .733944

Std. dev.: .22724

Percentiles: 10% 25% 50% 75% 90%

.428571 .6 .833333 1 1

--------------------------------------------------------------------------------------------------------------------------------------------------------------------------------

YearsFromGraduation

Medical school duration minus respondent's year in medical school (Response to question “Which year of medical school are you in?” minus response to question “What number of years is your medical school curriculum”)

--------------------------------------------------------------------------------------------------------------------------------------------------------------------------------

Type: Numeric (float)

Range: [0,5] Units: 1

Unique values: 6 Missing .: 11/908

Tabulation: Freq. Value

237 0

232 1

206 2

130 3

57 4

35 5

11 .

--------------------------------------------------------------------------------------------------------------------------------------------------------------------------------

Last2yearsMedSchool

Whether respondent is in last two years of medical school. If (Response to question “Which year of medical school are you in?” minus response to question “What number of years is your medical school curriculum”) is 0 or 1, then Last2yearsMedSchool = 1= in last two years of medical school, 0= earlier in medical school

--------------------------------------------------------------------------------------------------------------------------------------------------------------------------------

Type: Numeric (float)

Range: [0,1] Units: 1

Unique values: 2 Missing .: 11/908

Tabulation: Freq. Value Lable

428 0 Not last two years of medical school

469 1 In last two years of medical school

11 . Missing value

# Clinical learning evaluation questionnaire (CLEQ) motivation to learn subscale

## Raw data

--------------------------------------------------------------------------------------------------------------------------------------------------------------------------------

CLEQ1

Response to: Indicate how much you agree with the following statements: I am eager to learn

--------------------------------------------------------------------------------------------------------------------------------------------------------------------------------

Type: String (str17)

Unique values: 5 Missing "": 1/908

Tabulation: Freq. Value

1 ""

268 "Agree"

7 "Disagree"

612 "Strongly agree"

3 "Strongly disagree"

17 "Undecided"

--------------------------------------------------------------------------------------------------------------------------------------------------------------------------------

CLEQ2

Response to: Indicate how much you agree with the following statements: I am able to look for new information

--------------------------------------------------------------------------------------------------------------------------------------------------------------------------------

Type: String (str17)

Unique values: 5 Missing "": 1/908

Tabulation: Freq. Value

1 ""

410 "Agree"

8 "Disagree"

463 "Strongly agree"

1 "Strongly disagree"

25 "Undecided"

--------------------------------------------------------------------------------------------------------------------------------------------------------------------------------

CLEQ3

Response to: Indicate how much you agree with the following statements: I come to clinical sessions prepared and ready

--------------------------------------------------------------------------------------------------------------------------------------------------------------------------------

Type: String (str17)

Unique values: 5 Missing "": 0/908

Tabulation: Freq. Value

473 "Agree"

93 "Disagree"

101 "Strongly agree"

9 "Strongly disagree"

232 "Undecided"

--------------------------------------------------------------------------------------------------------------------------------------------------------------------------------

CLEQ4

Response to: Indicate how much you agree with the following statements: I enjoy learning in clinical sessions

--------------------------------------------------------------------------------------------------------------------------------------------------------------------------------

Type: String (str17)

Unique values: 5 Missing "": 1/908

Tabulation: Freq. Value

1 ""

334 "Agree"

13 "Disagree"

522 "Strongly agree"

3 "Strongly disagree"

35 "Undecided"

--------------------------------------------------------------------------------------------------------------------------------------------------------------------------------

CLEQ5

Response to: Indicate how much you agree with the following statements: I am able to express myself and show confidence

--------------------------------------------------------------------------------------------------------------------------------------------------------------------------------

Type: String (str17)

Unique values: 5 Missing "": 0/908

Tabulation: Freq. Value

391 "Agree"

104 "Disagree"

172 "Strongly agree"

18 "Strongly disagree"

223 "Undecided"

## Transformed data

--------------------------------------------------------------------------------------------------------------------------------------------------------------------------------

CLEQ1_n

CLEQ1 with addition of numeric value for each response to “Indicate how much you agree with the following statements: I am eager to learn”

--------------------------------------------------------------------------------------------------------------------------------------------------------------------------------

Type: Numeric (long)

Label: CLEQ_o

Range: [1,5] Units: 1

Unique values: 5 Missing .: 1/908

Tabulation: Freq. Numeric Label

3 1 Strongly disagree

7 2 Disagree

17 3 Undecided

268 4 Agree

612 5 Strongly agree

1 . Missing value

--------------------------------------------------------------------------------------------------------------------------------------------------------------------------------

CLEQ2_n

CLEQ2 with addition of numeric value for each response to “Indicate how much you agree with the following statements: I am able to look for new information”

--------------------------------------------------------------------------------------------------------------------------------------------------------------------------------

Type: Numeric (long)

Label: CLEQ_o

Range: [1,5] Units: 1

Unique values: 5 Missing .: 1/908

Tabulation: Freq. Numeric Label

1 1 Strongly disagree

8 2 Disagree

25 3 Undecided

410 4 Agree

463 5 Strongly agree

1 . Missing value

--------------------------------------------------------------------------------------------------------------------------------------------------------------------------------

CLEQ3_n

CLEQ3 with addition of numeric value for each response to “Indicate how much you agree with the following statements: I come to clinical sessions prepared and ready”

--------------------------------------------------------------------------------------------------------------------------------------------------------------------------------

Type: Numeric (long)

Label: CLEQ_o

Range: [1,5] Units: 1

Unique values: 5 Missing .: 0/908

Tabulation: Freq. Numeric Label

9 1 Strongly disagree

93 2 Disagree

232 3 Undecided

473 4 Agree

101 5 Strongly agree

--------------------------------------------------------------------------------------------------------------------------------------------------------------------------------

CLEQ4_n

CLEQ4 with addition of numeric value for each response to “Indicate how much you agree with the following statements: I enjoy learning in clinical sessions”

--------------------------------------------------------------------------------------------------------------------------------------------------------------------------------

Type: Numeric (long)

Label: CLEQ_o

Range: [1,5] Units: 1

Unique values: 5 Missing .: 1/908

Tabulation: Freq. Numeric Label

3 1 Strongly disagree

13 2 Disagree

35 3 Undecided

334 4 Agree

522 5 Strongly agree

1 . Missing value

--------------------------------------------------------------------------------------------------------------------------------------------------------------------------------

CLEQ5_n

CLEQ5 with addition of numeric value for each response to “Indicate how much you agree with the following statements: I am able to express myself and show confidence”

--------------------------------------------------------------------------------------------------------------------------------------------------------------------------------

Type: Numeric (long)

Label: CLEQ_o

Range: [1,5] Units: 1

Unique values: 5 Missing .: 0/908

Tabulation: Freq. Numeric Label

18 1 Strongly disagree

104 2 Disagree

223 3 Undecided

391 4 Agree

172 5 Strongly agree

--------------------------------------------------------------------------------------------------------------------------------------------------------------------------------

ExpressMyselfConfidence

RECODE of CLEQ5_n (I am able to express myself and show confidence), where those who strongly agreed or agreed are given value 1 “Agree”, while those who were undecided, diagreed or strongly disagreed were given value 0 “Not agree”

--------------------------------------------------------------------------------------------------------------------------------------------------------------------------------

Type: Numeric (long)

Label: ExpressMyselfConfidence

Range: [0,1] Units: 1

Unique values: 2 Missing .: 0/908

Tabulation: Freq. Numeric Label

345 0 Not Agree

563 1 Agree

# What happened during clinical supervision

## Raw data

--------------------------------------------------------------------------------------------------------------------------------------------------------------------------------

takethehistory

Answer to the question: Did you take the history?

--------------------------------------------------------------------------------------------------------------------------------------------------------------------------------

Type: String (str15)

Unique values: 4 Missing "": 0/908

Tabulation: Freq. Value

11 "Do not remember"

141 "No"

22 "Not applicable"

734 "Yes"

--------------------------------------------------------------------------------------------------------------------------------------------------------------------------------

doaclinicalexamination

Answer to the question: Did you do a clinical examination?

--------------------------------------------------------------------------------------------------------------------------------------------------------------------------------

Type: String (str15)

Unique values: 4 Missing "": 0/908

Tabulation: Freq. Value

8 "Do not remember"

136 "No"

23 "Not applicable"

741 "Yes"

--------------------------------------------------------------------------------------------------------------------------------------------------------------------------------

watchthesupervisortakethe

Answer to the question: Did you watch the supervisor take the history?

--------------------------------------------------------------------------------------------------------------------------------------------------------------------------------

Type: String (str15)

Unique values: 4 Missing "": 4/908

Tabulation: Freq. Value

4 ""

25 "Do not remember"

370 "No"

35 "Not applicable"

474 "Yes"

--------------------------------------------------------------------------------------------------------------------------------------------------------------------------------

watchthesupervisordoacli

Answer to the question: Did you watch the supervisor do a clinical examination?

--------------------------------------------------------------------------------------------------------------------------------------------------------------------------------

Type: String (str15)

Unique values: 4 Missing "": 1/908

Tabulation: Freq. Value

1 ""

17 "Do not remember"

294 "No"

27 "Not applicable"

569 "Yes"

--------------------------------------------------------------------------------------------------------------------------------------------------------------------------------

watchanotherstudenttaketh

Answer to the question: Did you watch another student take the history?

--------------------------------------------------------------------------------------------------------------------------------------------------------------------------------

Type: String (str15)

Unique values: 4 Missing "": 1/908

Tabulation: Freq. Value

1 ""

13 "Do not remember"

368 "No"

45 "Not applicable"

481 "Yes"

--------------------------------------------------------------------------------------------------------------------------------------------------------------------------------

watchanotherstudentdoacl

Answer to the question: Did you watch another student do a clinical examination?

--------------------------------------------------------------------------------------------------------------------------------------------------------------------------------

Type: String (str15)

Unique values: 4 Missing "": 3/908

Tabulation: Freq. Value

3 ""

12 "Do not remember"

376 "No"

47 "Not applicable"

470 "Yes"

Warning: Variable has embedded blanks.

--------------------------------------------------------------------------------------------------------------------------------------------------------------------------------

Forhowmuchofthetimeyouwer

Answer to the question: For how much of the time you were with the patient was the supervisor present?

--------------------------------------------------------------------------------------------------------------------------------------------------------------------------------

Type: String (str25)

Unique values: 4 Missing "": 2/908

Tabulation: Freq. Value

2 ""

233 "50% or more of the time"

295 "All the time"

275 "Less than 50% of the time"

103 "Not at all"

--------------------------------------------------------------------------------------------------------------------------------------------------------------------------------

Forwhichpartsofthepatient

Whether respondent tick off for “the beginning” in response to the question: For which part(s) of the patient encounter was the supervisor present?_1

--------------------------------------------------------------------------------------------------------------------------------------------------------------------------------

Type: String (str13)

Unique values: 1 Missing "": 368/908

Tabulation: Freq. Value

368 ""

540 "The beginning"

--------------------------------------------------------------------------------------------------------------------------------------------------------------------------------

U

Whether respondent tick off for “in the middle” in response to the question: For which part(s) of the patient encounter was the supervisor present?_2

--------------------------------------------------------------------------------------------------------------------------------------------------------------------------------

Type: String (str13)

Unique values: 1 Missing "": 505/908

Tabulation: Freq. Value

505 ""

403 "In the middle"

--------------------------------------------------------------------------------------------------------------------------------------------------------------------------------

V

Whether respondent tick off for “at the end” in response to the question: For which part(s) of the patient encounter was the supervisor present?_3

--------------------------------------------------------------------------------------------------------------------------------------------------------------------------------

Type: String (str10)

Unique values: 1 Missing "": 289/908

Tabulation: Freq. Value

289 ""

619 "At the end"

--------------------------------------------------------------------------------------------------------------------------------------------------------------------------------

W

Whether respondent tick off for “not applicable” in response to the question: For which part(s) of the patient encounter was the supervisor present?_4

--------------------------------------------------------------------------------------------------------------------------------------------------------------------------------

Type: String (str14)

Unique values: 1 Missing "": 760/908

Tabulation: Freq. Value

760 ""

148 "Not applicable"

--------------------------------------------------------------------------------------------------------------------------------------------------------------------------------

INCLUDINGYOURSELFhowmanystu

Response to the question “INCLUDING YOURSELF, how many students were present?”

--------------------------------------------------------------------------------------------------------------------------------------------------------------------------------

Type: String (str3)

Unique values: 20 Missing "": 16/908

Response option: Free text

--------------------------------------------------------------------------------------------------------------------------------------------------------------------------------

TakenCare

Response to the question: To what extent do you agree with the following statement: The patient was well taken care of

--------------------------------------------------------------------------------------------------------------------------------------------------------------------------------

Type: String (str26)

Unique values: 6 Missing "": 2/908

Tabulation: Freq. Value

2 ""

519 "Fully agree"

24 "Fully disagree"

37 "Neither disagree nor agree"

13 "Neither disagree or agree"

274 "Somewhat agree"

39 "Somewhat disagree"

--------------------------------------------------------------------------------------------------------------------------------------------------------------------------------

MyPerformance Response to the statement “The supervisor and I/the group discussed my performance”

--------------------------------------------------------------------------------------------------------------------------------------------------------------------------------

Type: String (str15)

Unique values: 4 Missing "": 2/908

Tabulation: Freq. Value

2 ""

47 "Do not remember"

362 "No"

45 "Not relevant"

452 "Yes"

--------------------------------------------------------------------------------------------------------------------------------------------------------------------------------

StudPerformance

Response to the statement “The supervisor and I/the group another student’s performance”

--------------------------------------------------------------------------------------------------------------------------------------------------------------------------------

Type: String (str15)

Unique values: 4 Missing "": 2/908

Tabulation: Freq. Value

2 ""

43 "Do not remember"

453 "No"

113 "Not relevant"

297 "Yes"

--------------------------------------------------------------------------------------------------------------------------------------------------------------------------------

Interviewing

Response to the statement “The supervisor and I/the group how to interview the patient”

--------------------------------------------------------------------------------------------------------------------------------------------------------------------------------

Type: String (str15)

Unique values: 4 Missing "": 1/908

Tabulation: Freq. Value

1 ""

34 "Do not remember"

276 "No"

30 "Not relevant"

567 "Yes"

--------------------------------------------------------------------------------------------------------------------------------------------------------------------------------

DiscussExamination

Response to the statement “The supervisor and I/the group how to examine the patient”

--------------------------------------------------------------------------------------------------------------------------------------------------------------------------------

Type: String (str15)

Unique values: 4 Missing "": 2/908

Tabulation: Freq. Value

2 ""

25 "Do not remember"

193 "No"

29 "Not relevant"

659 "Yes"

--------------------------------------------------------------------------------------------------------------------------------------------------------------------------------

ReportPatient

Response to the statement “The supervisor and I/the group how to present the patient’s history”

--------------------------------------------------------------------------------------------------------------------------------------------------------------------------------

Type: String (str15)

Unique values: 4 Missing "": 1/908

Tabulation: Freq. Value

1 ""

31 "Do not remember"

373 "No"

36 "Not relevant"

467 "Yes"

--------------------------------------------------------------------------------------------------------------------------------------------------------------------------------

DiscussTP

Response to the statement “The supervisor and I/the group tests and/or procedures”

--------------------------------------------------------------------------------------------------------------------------------------------------------------------------------

Type: String (str15)

Unique values: 4 Missing "": 1/908

Tabulation: Freq. Value

1 ""

53 "Do not remember"

253 "No"

27 "Not relevant"

574 "Yes"

--------------------------------------------------------------------------------------------------------------------------------------------------------------------------------

DifferentialDiagnosis Response to the statement “The supervisor and I/the group differential diagnosis”

--------------------------------------------------------------------------------------------------------------------------------------------------------------------------------

Type: String (str15)

Unique values: 4 Missing "": 5/908

Tabulation: Freq. Value

5 ""

46 "Do not remember"

180 "No"

20 "Not relevant"

657 "Yes"

--------------------------------------------------------------------------------------------------------------------------------------------------------------------------------

DiscussMP Response to the statement “The supervisor and I/the group treatment plan(s)”

--------------------------------------------------------------------------------------------------------------------------------------------------------------------------------

Type: String (str15)

Unique values: 4 Missing "": 3/908

Tabulation: Freq. Value

3 ""

43 "Do not remember"

197 "No"

16 "Not relevant"

649 "Yes"

--------------------------------------------------------------------------------------------------------------------------------------------------------------------------------

professionalism Response to the statement “The supervisor and I/the group professionalism”

--------------------------------------------------------------------------------------------------------------------------------------------------------------------------------

Type: String (str15)

Unique values: 4 Missing "": 4/908

Tabulation: Freq. Value

4 ""

76 "Do not remember"

385 "No"

20 "Not relevant"

423 "Yes"

--------------------------------------------------------------------------------------------------------------------------------------------------------------------------------

otherpleasespecity Response to the statement “The supervisor and I/the group other discussed other, please specity”

--------------------------------------------------------------------------------------------------------------------------------------------------------------------------------

Type: String (str15)

Unique values: 4 Missing "": 96/908

Tabulation: Freq. Value

96 ""

181 "Do not remember"

356 "No"

225 "Not relevant"

50 "Yes"

--------------------------------------------------------------------------------------------------------------------------------------------------------------------------------

Other Respondents free text responses to what non-listed topics they discussed with their supervisor

--------------------------------------------------------------------------------------------------------------------------------------------------------------------------------

Type: String (str587)

Unique values: 42 Missing "": 866/908

## Transformed data

--------------------------------------------------------------------------------------------------------------------------------------------------------------------------------

DegreeofSupervisorPresence

Adding numeric values to the response to the questions “For how much of the time you were with the patient was the supervisor present?”

--------------------------------------------------------------------------------------------------------------------------------------------------------------------------------

Type: Numeric (long)

Label: DegreeofSupervisorPresence

Range: [1,4] Units: 1

Unique values: 4 Missing .: 2/908

Tabulation: Freq. Numeric Label

233 1 50% or more of the time

295 2 All the time

275 3 Less than 50% of the time

103 4 Not at all

2 . Missing value

--------------------------------------------------------------------------------------------------------------------------------------------------------------------------------

SupervisorPresence

Estimate of percentage of time of supervisor presence based on response to question “For how much of the time you were with the patient was the supervisor present?”

--------------------------------------------------------------------------------------------------------------------------------------------------------------------------------

Type: Numeric (long)

Label: SupPres

Range: [0,100] Units: 1

Unique values: 4 Missing .: 2/908

Tabulation: Freq. Numeric Label

103 0 Not at all

275 25 Less than 50% of the time

233 75 50% or more of the time

295 100 All the time

2 . Missing value

--------------------------------------------------------------------------------------------------------------------------------------------------------------------------------

TheBeginning

Whether respondent tick off for “the beginning” in response to the question: “For which part(s) of the patient encounter was the supervisor present?” with addition of numeric values

--------------------------------------------------------------------------------------------------------------------------------------------------------------------------------

Type: Numeric (long)

Label: TheBeginning

Range: [1,1] Units: 1

Unique values: 1 Missing .: 368/908

Tabulation: Freq. Numeric Label

540 1 The beginning

368 . Missing value/not ticked this box

--------------------------------------------------------------------------------------------------------------------------------------------------------------------------------

InTheMiddle

Whether respondent tick off for “in the middle” in response to the question: “For which part(s) of the patient encounter was the supervisor present?” with addition of numeric values

--------------------------------------------------------------------------------------------------------------------------------------------------------------------------------

Type: Numeric (long)

Label: InTheMiddle

Range: [1,1] Units: 1

Unique values: 1 Missing .: 505/908

Tabulation: Freq. Numeric Label

403 1 In the middle

505 . Missing value/not ticked this box

--------------------------------------------------------------------------------------------------------------------------------------------------------------------------------

TheEnd

Whether respondent tick off for “the end” in response to the question: “For which part(s) of the patient encounter was the supervisor present?” with addition of numeric values

--------------------------------------------------------------------------------------------------------------------------------------------------------------------------------

Type: Numeric (long)

Label: TheEnd

Range: [1,1] Units: 1

Unique values: 1 Missing .: 289/908

Tabulation: Freq. Numeric Label

619 1 At the end

289 . Missing value/not ticked this box

--------------------------------------------------------------------------------------------------------------------------------------------------------------------------------

NotApplicable

Whether respondent tick off for “not applicable” in response to the question: “For which part(s) of the patient encounter was the supervisor present?” with addition of numeric values

--------------------------------------------------------------------------------------------------------------------------------------------------------------------------------

Type: Numeric (long)

Label: NotApplicable

Range: [1,1] Units: 1

Unique values: 1 Missing .: 760/908

Tabulation: Freq. Numeric Label

148 1 Not applicable

760 . Missing value/not ticked this box

--------------------------------------------------------------------------------------------------------------------------------------------------------------------------------

TakenCaren_n

RECODE of TakenCare_n (Response to the question: “To what extent do you agree with the following statement: The patient was well taken care of”) with addition of values from disagreeing the most (1) to agreeing the most (5), combining “Neither disagree or agree” and “Neither disagree nor agree” (of which one was used in the survey to Norwegian medical students abroad and the other in the other survey).

--------------------------------------------------------------------------------------------------------------------------------------------------------------------------------

Type: Numeric (long)

Label: TakenCaren_n

Range: [1,5] Units: 1

Unique values: 5 Missing .: 2/908

Tabulation: Freq. Numeric Label

24 1 Fully disagree

39 2 Somewhat disagree

50 3 Neither disagree nor agree

274 4 Somewhat agree

519 5 Fully agree

2 .

.

--------------------------------------------------------------------------------------------------------------------------------------------------------------------------------

StudentsPresent

Destringing “INCLUDING YOURSELF, how many students were present?” so that a numerical value is given for the answer

--------------------------------------------------------------------------------------------------------------------------------------------------------------------------------

Type: Numeric (long)

Label: StudentsPresent

Range: [1,20] Units: 1

Unique values: 20 Missing .: 16/908

--------------------------------------------------------------------------------------------------------------------------------------------------------------------------------

PeerSupport

RECODE of StudentsPresent

Response to: “INCLUDING YOURSELF, how many students were present?” grouped into only responding student and more students than just the responding students

--------------------------------------------------------------------------------------------------------------------------------------------------------------------------------

Type: Numeric (long)

Range: [0,1] Units: 1

Unique values: 2 Missing .: 16/908

Tabulation: Freq. Value Label

284 0 Only responding student

608 1 At least one student in addition to responding student

16 . Missing values

# Maastricht Clinical Teaching Questionnaire

## Raw data

--------------------------------------------------------------------------------------------------------------------------------------------------------------------------------

MCTQ1

Response to: Indicate how much you agree with the following statements: The clinical supervisor Consistently demonstrated how to perform clinical skills

--------------------------------------------------------------------------------------------------------------------------------------------------------------------------------

Type: String (str26)

Unique values: 6 Missing "": 4/908

Tabulation: Freq. Value

4 ""

170 "Fully agree"

82 "Fully disagree"

37 "Neither agree nor disagree"

121 "Neither disagree nor agree"

327 "Somewhat agree"

167 "Somewhat disagree"

--------------------------------------------------------------------------------------------------------------------------------------------------------------------------------

MCTQ2

Response to: Indicate how much you agree with the following statements: The clinical supervisor Created sufficient opportunities for me to observe him/her

--------------------------------------------------------------------------------------------------------------------------------------------------------------------------------

Type: String (str26)

Unique values: 6 Missing "": 7/908

Tabulation: Freq. Value

7 ""

271 "Fully agree"

68 "Fully disagree"

26 "Neither agree nor disagree"

105 "Neither disagree nor agree"

294 "Somewhat agree"

137 "Somewhat disagree"

--------------------------------------------------------------------------------------------------------------------------------------------------------------------------------

MCTQ3

Response to: Indicate how much you agree with the following statements: The clinical supervisor Served as a role model as to the kind of doctor I would like to become

--------------------------------------------------------------------------------------------------------------------------------------------------------------------------------

Type: String (str26)

Unique values: 6 Missing "": 5/908

Tabulation: Freq. Value

5 ""

265 "Fully agree"

65 "Fully disagree"

47 "Neither agree nor disagree"

136 "Neither disagree nor agree"

286 "Somewhat agree"

104 "Somewhat disagree"

--------------------------------------------------------------------------------------------------------------------------------------------------------------------------------

MCTQ4

Response to: Indicate how much you agree with the following statements: The clinical supervisor Gave useful feedback during or immediately after direct observation of my patient encounters

--------------------------------------------------------------------------------------------------------------------------------------------------------------------------------

Type: String (str26)

Unique values: 6 Missing "": 6/908

Tabulation: Freq. Value

6 ""

265 "Fully agree"

86 "Fully disagree"

27 "Neither agree nor disagree"

107 "Neither disagree nor agree"

309 "Somewhat agree"

108 "Somewhat disagree"

--------------------------------------------------------------------------------------------------------------------------------------------------------------------------------

MCTQ5

Response to: Indicate how much you agree with the following statements: The clinical supervisor Adjusted his/her teaching to my level of experience

--------------------------------------------------------------------------------------------------------------------------------------------------------------------------------

Type: String (str26)

Unique values: 6 Missing "": 5/908

Tabulation: Freq. Value

5 ""

309 "Fully agree"

61 "Fully disagree"

36 "Neither agree nor disagree"

99 "Neither disagree nor agree"

310 "Somewhat agree"

88 "Somewhat disagree"

--------------------------------------------------------------------------------------------------------------------------------------------------------------------------------

MCTQ6

Response to: Indicate how much you agree with the following statements: The clinical supervisor Offered me sufficient opportunities to perform activities independently

--------------------------------------------------------------------------------------------------------------------------------------------------------------------------------

Type: String (str26)

Unique values: 6 Missing "": 5/908

Tabulation: Freq. Value

5 ""

327 "Fully agree"

72 "Fully disagree"

29 "Neither agree nor disagree"

92 "Neither disagree nor agree"

277 "Somewhat agree"

106 "Somewhat disagree"

--------------------------------------------------------------------------------------------------------------------------------------------------------------------------------

MCTQ7

Response to: Indicate how much you agree with the following statements: The clinical supervisor Asked me to provide rational for my actions

--------------------------------------------------------------------------------------------------------------------------------------------------------------------------------

Type: String (str26)

Unique values: 6 Missing "": 7/908

Tabulation: Freq. Value

7 ""

188 "Fully agree"

71 "Fully disagree"

73 "Neither agree nor disagree"

199 "Neither disagree nor agree"

272 "Somewhat agree"

98 "Somewhat disagree"

--------------------------------------------------------------------------------------------------------------------------------------------------------------------------------

MCTQ8

Response to: Indicate how much you agree with the following statements: The clinical supervisor Asked me questions aimed at increasing my understanding

--------------------------------------------------------------------------------------------------------------------------------------------------------------------------------

Type: String (str26)

Unique values: 6 Missing "": 4/908

Tabulation: Freq. Value

4 ""

285 "Fully agree"

44 "Fully disagree"

25 "Neither agree nor disagree"

93 "Neither disagree nor agree"

371 "Somewhat agree"

86 "Somewhat disagree"

--------------------------------------------------------------------------------------------------------------------------------------------------------------------------------

MCTQ9

Response to: Indicate how much you agree with the following statements: The clinical supervisor Stimulated me to explore my strengths and weaknesses

--------------------------------------------------------------------------------------------------------------------------------------------------------------------------------

Type: String (str26)

Unique values: 6 Missing "": 4/908

Tabulation: Freq. Value

4 ""

135 "Fully agree"

112 "Fully disagree"

80 "Neither agree nor disagree"

177 "Neither disagree nor agree"

218 "Somewhat agree"

182 "Somewhat disagree"

--------------------------------------------------------------------------------------------------------------------------------------------------------------------------------

MCTQ10

Response to: Indicate how much you agree with the following statements: The clinical supervisor Encouraged me to formulate learning goals

--------------------------------------------------------------------------------------------------------------------------------------------------------------------------------

Type: String (str26)

Unique values: 6 Missing "": 7/908

Tabulation: Freq. Value

7 ""

84 "Fully agree"

234 "Fully disagree"

72 "Neither agree nor disagree"

175 "Neither disagree nor agree"

125 "Somewhat agree"

211 "Somewhat disagree"

--------------------------------------------------------------------------------------------------------------------------------------------------------------------------------

MCTQ11

Response to: Indicate how much you agree with the following statements: The clinical supervisor Encouraged me to pursue my learning goals

--------------------------------------------------------------------------------------------------------------------------------------------------------------------------------

Type: String (str26)

Unique values: 6 Missing "": 7/908

Tabulation: Freq. Value

7 ""

116 "Fully agree"

169 "Fully disagree"

64 "Neither agree nor disagree"

199 "Neither disagree nor agree"

201 "Somewhat agree"

152 "Somewhat disagree"

--------------------------------------------------------------------------------------------------------------------------------------------------------------------------------

MCTQ12

Response to: Indicate how much you agree with the following statements: The clinical supervisor Created a safe learning environment

--------------------------------------------------------------------------------------------------------------------------------------------------------------------------------

Type: String (str26)

Unique values: 6 Missing "": 4/908

Tabulation: Freq. Value

4 ""

330 "Fully agree"

56 "Fully disagree"

38 "Neither agree nor disagree"

125 "Neither disagree nor agree"

289 "Somewhat agree"

66 "Somewhat disagree"

--------------------------------------------------------------------------------------------------------------------------------------------------------------------------------

MCTQ13

Response to: Indicate how much you agree with the following statements: The clinical supervisor Was genuinely interested in me as a student

--------------------------------------------------------------------------------------------------------------------------------------------------------------------------------

Type: String (str26)

Unique values: 6 Missing "": 5/908

Tabulation: Freq. Value

5 ""

231 "Fully agree"

110 "Fully disagree"

54 "Neither agree nor disagree"

143 "Neither disagree nor agree"

247 "Somewhat agree"

118 "Somewhat disagree"

--------------------------------------------------------------------------------------------------------------------------------------------------------------------------------

MCTQ14

Response to: Indicate how much you agree with the following statements: The clinical supervisor Showed that he/she respected me

--------------------------------------------------------------------------------------------------------------------------------------------------------------------------------

Type: String (str26)

Unique values: 6 Missing "": 4/908

Tabulation: Freq. Value

4 ""

380 "Fully agree"

54 "Fully disagree"

48 "Neither agree nor disagree"

116 "Neither disagree nor agree"

239 "Somewhat agree"

67 "Somewhat disagree"

## Transformed data

--------------------------------------------------------------------------------------------------------------------------------------------------------------------------------

MCTQ1_n

MCTQ1 with addition of numeric value for each response to “Indicate how much you agree with the following statements: The clinical supervisor Consistently demonstrated how to perform clinical skills”

--------------------------------------------------------------------------------------------------------------------------------------------------------------------------------

Type: Numeric (long)

Label: MCTQ_o

Range: [1,6] Units: 1

Unique values: 6 Missing .: 4/908

Tabulation: Freq. Numeric Label

82 1 Fully disagree

167 2 Somewhat disagree

37 3 Neither agree nor disagree

327 4 Somewhat agree

170 5 Fully agree

121 6 Neither disagree nor agree

4 . Missing value

--------------------------------------------------------------------------------------------------------------------------------------------------------------------------------

MCTQ2_n

MCTQ2 with addition of numeric value for each response to “Indicate how much you agree with the following statements: The clinical supervisor Created sufficient opportunities for me to observe him/her”

--------------------------------------------------------------------------------------------------------------------------------------------------------------------------------

Type: Numeric (long)

Label: MCTQ_o

Range: [1,6] Units: 1

Unique values: 6 Missing .: 7/908

Tabulation: Freq. Numeric Label

68 1 Fully disagree

137 2 Somewhat disagree

26 3 Neither agree nor disagree

294 4 Somewhat agree

271 5 Fully agree

105 6 Neither disagree nor agree

7 . Missing value

--------------------------------------------------------------------------------------------------------------------------------------------------------------------------------

MCTQ3_n

MCTQ3 with addition of numeric value for each response to “Indicate how much you agree with the following statements: The clinical supervisor Served as a role model as to the kind of doctor I would like to become”

--------------------------------------------------------------------------------------------------------------------------------------------------------------------------------

Type: Numeric (long)

Label: MCTQ_o

Range: [1,6] Units: 1

Unique values: 6 Missing .: 5/908

Tabulation: Freq. Numeric Label

65 1 Fully disagree

104 2 Somewhat disagree

47 3 Neither agree nor disagree

286 4 Somewhat agree

265 5 Fully agree

136 6 Neither disagree nor agree

5 . Missing value

--------------------------------------------------------------------------------------------------------------------------------------------------------------------------------

MCTQ4_n

MCTQ4 with addition of numeric value for each response to “Indicate how much you agree with the following statements: The clinical supervisor Gave useful feedback during or immediately after direct observation of my patient encounters”

--------------------------------------------------------------------------------------------------------------------------------------------------------------------------------

Type: Numeric (long)

Label: MCTQ_o

Range: [1,6] Units: 1

Unique values: 6 Missing .: 6/908

Tabulation: Freq. Numeric Label

86 1 Fully disagree

108 2 Somewhat disagree

27 3 Neither agree nor disagree

309 4 Somewhat agree

265 5 Fully agree

107 6 Neither disagree nor agree

6 . Missing value

--------------------------------------------------------------------------------------------------------------------------------------------------------------------------------

MCTQ5_n

MCTQ5 with addition of numeric value for each response to “Indicate how much you agree with the following statements: The clinical supervisor Adjusted his/her teaching to my level of experience”

--------------------------------------------------------------------------------------------------------------------------------------------------------------------------------

Type: Numeric (long)

Label: MCTQ_o

Range: [1,6] Units: 1

Unique values: 6 Missing .: 5/908

Tabulation: Freq. Numeric Label

61 1 Fully disagree

88 2 Somewhat disagree

36 3 Neither agree nor disagree

310 4 Somewhat agree

309 5 Fully agree

99 6 Neither disagree nor agree

5 . Missing value

--------------------------------------------------------------------------------------------------------------------------------------------------------------------------------

MCTQ6_n

MCTQ6 with addition of numeric value for each response to “Indicate how much you agree with the following statements: The clinical supervisor Offered me sufficient opportunities to perform activities independently”

--------------------------------------------------------------------------------------------------------------------------------------------------------------------------------

Type: Numeric (long)

Label: MCTQ_o

Range: [1,6] Units: 1

Unique values: 6 Missing .: 5/908

Tabulation: Freq. Numeric Label

72 1 Fully disagree

106 2 Somewhat disagree

29 3 Neither agree nor disagree

277 4 Somewhat agree

327 5 Fully agree

92 6 Neither disagree nor agree

5 . Missing value

--------------------------------------------------------------------------------------------------------------------------------------------------------------------------------

MCTQ7_n

MCTQ7 with addition of numeric value for each response to “Indicate how much you agree with the following statements: The clinical supervisor Asked me to provide rational for my actions”

--------------------------------------------------------------------------------------------------------------------------------------------------------------------------------

Type: Numeric (long)

Label: MCTQ_o

Range: [1,6] Units: 1

Unique values: 6 Missing .: 7/908

Tabulation: Freq. Numeric Label

71 1 Fully disagree

98 2 Somewhat disagree

73 3 Neither agree nor disagree

272 4 Somewhat agree

188 5 Fully agree

199 6 Neither disagree nor agree

7 . Missing value

--------------------------------------------------------------------------------------------------------------------------------------------------------------------------------

MCTQ8_n

MCTQ8 with addition of numeric value for each response to “Indicate how much you agree with the following statements: The clinical supervisor Asked me questions aimed at increasing my understanding”

--------------------------------------------------------------------------------------------------------------------------------------------------------------------------------

Type: Numeric (long)

Label: MCTQ_o

Range: [1,6] Units: 1

Unique values: 6 Missing .: 4/908

Tabulation: Freq. Numeric Label

44 1 Fully disagree

86 2 Somewhat disagree

25 3 Neither agree nor disagree

371 4 Somewhat agree

285 5 Fully agree

93 6 Neither disagree nor agree

4 . Missing value

--------------------------------------------------------------------------------------------------------------------------------------------------------------------------------

MCTQ9_n

MCTQ9 with addition of numeric value for each response to “Indicate how much you agree with the following statements: The clinical supervisor Stimulated me to explore my strengths and weaknesses”

--------------------------------------------------------------------------------------------------------------------------------------------------------------------------------

Type: Numeric (long)

Label: MCTQ_o

Range: [1,6] Units: 1

Unique values: 6 Missing .: 4/908

Tabulation: Freq. Numeric Label

112 1 Fully disagree

182 2 Somewhat disagree

80 3 Neither agree nor disagree

218 4 Somewhat agree

135 5 Fully agree

177 6 Neither disagree nor agree

4 . Missing value

--------------------------------------------------------------------------------------------------------------------------------------------------------------------------------

MCTQ10_n

MCTQ10 with addition of numeric value for each response to “Indicate how much you agree with the following statements: The clinical supervisor Encouraged me to formulate learning goals”

--------------------------------------------------------------------------------------------------------------------------------------------------------------------------------

Type: Numeric (long)

Label: MCTQ_o

Range: [1,6] Units: 1

Unique values: 6 Missing .: 7/908

Tabulation: Freq. Numeric Label

234 1 Fully disagree

211 2 Somewhat disagree

72 3 Neither agree nor disagree

125 4 Somewhat agree

84 5 Fully agree

175 6 Neither disagree nor agree

7 . Missing value

--------------------------------------------------------------------------------------------------------------------------------------------------------------------------------

MCTQ11_n

MCTQ11 with addition of numeric value for each response to “Indicate how much you agree with the following statements: The clinical supervisor Encouraged me to pursue my learning goals”

--------------------------------------------------------------------------------------------------------------------------------------------------------------------------------

Type: Numeric (long)

Label: MCTQ_o

Range: [1,6] Units: 1

Unique values: 6 Missing .: 7/908

Tabulation: Freq. Numeric Label

169 1 Fully disagree

152 2 Somewhat disagree

64 3 Neither agree nor disagree

201 4 Somewhat agree

116 5 Fully agree

199 6 Neither disagree nor agree

7 . Missing value

--------------------------------------------------------------------------------------------------------------------------------------------------------------------------------

MCTQ12_n

MCTQ12 with addition of numeric value for each response to “Indicate how much you agree with the following statements: The clinical supervisor Created a safe learning environment”

--------------------------------------------------------------------------------------------------------------------------------------------------------------------------------

Type: Numeric (long)

Label: MCTQ_o

Range: [1,6] Units: 1

Unique values: 6 Missing .: 4/908

Tabulation: Freq. Numeric Label

56 1 Fully disagree

66 2 Somewhat disagree

38 3 Neither agree nor disagree

289 4 Somewhat agree

330 5 Fully agree

125 6 Neither disagree nor agree

4 . Missing value

--------------------------------------------------------------------------------------------------------------------------------------------------------------------------------

MCTQ13_n

MCTQ13 with addition of numeric value for each response to “Indicate how much you agree with the following statements: The clinical supervisor Was genuinely interested in me as a student”

--------------------------------------------------------------------------------------------------------------------------------------------------------------------------------

Type: Numeric (long)

Label: MCTQ_o

Range: [1,6] Units: 1

Unique values: 6 Missing .: 5/908

Tabulation: Freq. Numeric Label

110 1 Fully disagree

118 2 Somewhat disagree

54 3 Neither agree nor disagree

247 4 Somewhat agree

231 5 Fully agree

143 6 Neither disagree nor agree

5 . Missing value

--------------------------------------------------------------------------------------------------------------------------------------------------------------------------------

MCTQ14_n

MCTQ14 with addition of numeric value for each response to “Indicate how much you agree with the following statements: The clinical supervisor Showed that he/she respected me”

--------------------------------------------------------------------------------------------------------------------------------------------------------------------------------

Type: Numeric (long)

Label: MCTQ_o

Range: [1,6] Units: 1

Unique values: 6 Missing .: 4/908

Tabulation: Freq. Numeric Label

54 1 Fully disagree

67 2 Somewhat disagree

48 3 Neither agree nor disagree

239 4 Somewhat agree

380 5 Fully agree

116 6 Neither disagree nor agree

4 . Missing value

--------------------------------------------------------------------------------------------------------------------------------------------------------------------------------

MCTQ1n_n

RECODE of MCTQ1_n (response to “Indicate how much you agree with the following statements: The clinical supervisor Consistently demonstrated how to perform clinical skills”) so that “Neither agree nor disagree” and “Neither disagree nor agree” have the same number and is in the middle of the scale from “fully disagree” to “fully agree”

--------------------------------------------------------------------------------------------------------------------------------------------------------------------------------

Type: Numeric (long)

Label: MCTQ1n_n

Range: [1,5] Units: 1

Unique values: 5 Missing .: 4/908

Tabulation: Freq. Numeric Label

82 1 Fully disagree

167 2 Somewhat disagree

158 3 Neither disagree nor agree

327 4 Somewhat agree

170 5 Fully agree

4 . Missing value

--------------------------------------------------------------------------------------------------------------------------------------------------------------------------------

MCTQ2n_n

RECODE of MCTQ2_n (response to “Indicate how much you agree with the following statements: The clinical supervisor Created sufficient opportunities for me to observe him/her”) so that “Neither agree nor disagree” and “Neither disagree nor agree” have the same number and is in the middle of the scale from “fully disagree” to “fully agree”

--------------------------------------------------------------------------------------------------------------------------------------------------------------------------------

Type: Numeric (long)

Label: MCTQ2n_n

Range: [1,5] Units: 1

Unique values: 5 Missing .: 7/908

Tabulation: Freq. Numeric Label

68 1 Fully disagree

137 2 Somewhat disagree

131 3 Neither disagree nor agree

294 4 Somewhat agree

271 5 Fully agree

7 . Missing value

--------------------------------------------------------------------------------------------------------------------------------------------------------------------------------

MCTQ3n_n

RECODE of MCTQ3_n (response to “Indicate how much you agree with the following statements: The clinical supervisor Served as a role model as to the kind of doctor I would like to become”) so that “Neither agree nor disagree” and “Neither disagree nor agree” have the same number and is in the middle of the scale from “fully disagree” to “fully agree”

--------------------------------------------------------------------------------------------------------------------------------------------------------------------------------

Type: Numeric (long)

Label: MCTQ3n_n

Range: [1,5] Units: 1

Unique values: 5 Missing .: 5/908

Tabulation: Freq. Numeric Label

65 1 Fully disagree

104 2 Somewhat disagree

183 3 Neither disagree nor agree

286 4 Somewhat agree

265 5 Fully agree

5 . Missing value

--------------------------------------------------------------------------------------------------------------------------------------------------------------------------------

MCTQ4n_n

RECODE of MCTQ4_n (response to “Indicate how much you agree with the following statements: The clinical supervisor Gave useful feedback during or immediately after direct observation of my patient encounters”) so that “Neither agree nor disagree” and “Neither disagree nor agree” have the same number and is in the middle of the scale from “fully disagree” to “fully agree”

--------------------------------------------------------------------------------------------------------------------------------------------------------------------------------

Type: Numeric (long)

Label: MCTQ4n_n

Range: [1,5] Units: 1

Unique values: 5 Missing .: 6/908

Tabulation: Freq. Numeric Label

86 1 Fully disagree

108 2 Somewhat disagree

134 3 Neither disagree nor agree

309 4 Somewhat agree

265 5 Fully agree

6 . Missing value

--------------------------------------------------------------------------------------------------------------------------------------------------------------------------------

MCTQ5n_n

RECODE of MCTQ5_n (response to “Indicate how much you agree with the following statements: The clinical supervisor Adjusted his/her teaching to my level of experience”) so that “Neither agree nor disagree” and “Neither disagree nor agree” have the same number and is in the middle of the scale from “fully disagree” to “fully agree”

--------------------------------------------------------------------------------------------------------------------------------------------------------------------------------

Type: Numeric (long)

Label: MCTQ5n_n

Range: [1,5] Units: 1

Unique values: 5 Missing .: 5/908

Tabulation: Freq. Numeric Label

61 1 Fully disagree

88 2 Somewhat disagree

135 3 Neither disagree nor agree

310 4 Somewhat agree

309 5 Fully agree

5 . Missing value

--------------------------------------------------------------------------------------------------------------------------------------------------------------------------------

MCTQ6n_n

RECODE of MCTQ6_n (response to “Indicate how much you agree with the following statements: The clinical supervisor Offered me sufficient opportunities to perform activities independently”) so that “Neither agree nor disagree” and “Neither disagree nor agree” have the same number and is in the middle of the scale from “fully disagree” to “fully agree”

--------------------------------------------------------------------------------------------------------------------------------------------------------------------------------

Type: Numeric (long)

Label: MCTQ6n_n

Range: [1,5] Units: 1

Unique values: 5 Missing .: 5/908

Tabulation: Freq. Numeric Label

72 1 Fully disagree

106 2 Somewhat disagree

121 3 Neither disagree nor agree

277 4 Somewhat agree

327 5 Fully agree

5 . Missing value

--------------------------------------------------------------------------------------------------------------------------------------------------------------------------------

MCTQ7n_n

RECODE of MCTQ7_n (response to “Indicate how much you agree with the following statements: The clinical supervisor Asked me to provide rational for my actions”) so that “Neither agree nor disagree” and “Neither disagree nor agree” have the same number and is in the middle of the scale from “fully disagree” to “fully agree”

--------------------------------------------------------------------------------------------------------------------------------------------------------------------------------

Type: Numeric (long)

Label: MCTQ7n_n

Range: [1,5] Units: 1

Unique values: 5 Missing .: 7/908

Tabulation: Freq. Numeric Label

71 1 Fully disagree

98 2 Somewhat disagree

272 3 Neither disagree nor agree

272 4 Somewhat agree

188 5 Fully agree

7 . Missing value

--------------------------------------------------------------------------------------------------------------------------------------------------------------------------------

MCTQ8n_n

RECODE of MCTQ8_n (response to “Indicate how much you agree with the following statements: The clinical supervisor Asked me questions aimed at increasing my understanding”) so that “Neither agree nor disagree” and “Neither disagree nor agree” have the same number and is in the middle of the scale from “fully disagree” to “fully agree”

--------------------------------------------------------------------------------------------------------------------------------------------------------------------------------

Type: Numeric (long)

Label: MCTQ8n_n

Range: [1,5] Units: 1

Unique values: 5 Missing .: 4/908

Tabulation: Freq. Numeric Label

44 1 Fully disagree

86 2 Somewhat disagree

118 3 Neither disagree nor agree

371 4 Somewhat agree

285 5 Fully agree

4 . Missing value

--------------------------------------------------------------------------------------------------------------------------------------------------------------------------------

MCTQ9n_n

RECODE of MCTQ9_n (response to “Indicate how much you agree with the following statements: The clinical supervisor Stimulated me to explore my strengths and weaknesses”) so that “Neither agree nor disagree” and “Neither disagree nor agree” have the same number and is in the middle of the scale from “fully disagree” to “fully agree”

--------------------------------------------------------------------------------------------------------------------------------------------------------------------------------

Type: Numeric (long)

Label: MCTQ9n_n

Range: [1,5] Units: 1

Unique values: 5 Missing .: 4/908

Tabulation: Freq. Numeric Label

112 1 Fully disagree

182 2 Somewhat disagree

257 3 Neither disagree nor agree

218 4 Somewhat agree

135 5 Fully agree

4 . Missing value

--------------------------------------------------------------------------------------------------------------------------------------------------------------------------------

MCTQ10n_n

RECODE of MCTQ10_n (response to “Indicate how much you agree with the following statements: The clinical supervisor Encouraged me to formulate learning goals”) so that “Neither agree nor disagree” and “Neither disagree nor agree” have the same number and is in the middle of the scale from “fully disagree” to “fully agree”

--------------------------------------------------------------------------------------------------------------------------------------------------------------------------------

Type: Numeric (long)

Label: MCTQ10n_n

Range: [1,5] Units: 1

Unique values: 5 Missing .: 7/908

Tabulation: Freq. Numeric Label

234 1 Fully disagree

211 2 Somewhat disagree

247 3 Neither disagree nor agree

125 4 Somewhat agree

84 5 Fully agree

7 . Missing value

--------------------------------------------------------------------------------------------------------------------------------------------------------------------------------

MCTQ11n_n

RECODE of MCTQ11_n (response to “Indicate how much you agree with the following statements: The clinical supervisor Encouraged me to pursue my learning goals”) so that “Neither agree nor disagree” and “Neither disagree nor agree” have the same number and is in the middle of the scale from “fully disagree” to “fully agree”

--------------------------------------------------------------------------------------------------------------------------------------------------------------------------------

Type: Numeric (long)

Label: MCTQ11n_n

Range: [1,5] Units: 1

Unique values: 5 Missing .: 7/908

Tabulation: Freq. Numeric Label

169 1 Fully disagree

152 2 Somewhat disagree

263 3 Neither disagree nor agree

201 4 Somewhat agree

116 5 Fully agree

7 . Missing value

--------------------------------------------------------------------------------------------------------------------------------------------------------------------------------

MCTQ12n_n

RECODE of MCTQ12_n (C response to “Indicate how much you agree with the following statements: The clinical supervisor reated a safe learning environment”) so that “Neither agree nor disagree” and “Neither disagree nor agree” have the same number and is in the middle of the scale from “fully disagree” to “fully agree”

--------------------------------------------------------------------------------------------------------------------------------------------------------------------------------

Type: Numeric (long)

Label: MCTQ12n_n

Range: [1,5] Units: 1

Unique values: 5 Missing .: 4/908

Tabulation: Freq. Numeric Label

56 1 Fully disagree

66 2 Somewhat disagree

163 3 Neither disagree nor agree

289 4 Somewhat agree

330 5 Fully agree

4 . Missing value

--------------------------------------------------------------------------------------------------------------------------------------------------------------------------------

MCTQ13n_n

RECODE of MCTQ13_n (response to “Indicate how much you agree with the following statements: The clinical supervisor Was genuinely interested in me as a student”) so that “Neither agree nor disagree” and “Neither disagree nor agree” have the same number and is in the middle of the scale from “fully disagree” to “fully agree”

--------------------------------------------------------------------------------------------------------------------------------------------------------------------------------

Type: Numeric (long)

Label: MCTQ13n_n

Range: [1,5] Units: 1

Unique values: 5 Missing .: 5/908

Tabulation: Freq. Numeric Label

110 1 Fully disagree

118 2 Somewhat disagree

197 3 Neither disagree nor agree

247 4 Somewhat agree

231 5 Fully agree

5 . Missing value

--------------------------------------------------------------------------------------------------------------------------------------------------------------------------------

MCTQ14n_n

RECODE of MCTQ14_n (response to “Indicate how much you agree with the following statements: The clinical supervisor Showed that he/she respected me”) so that “Neither agree nor disagree” and “Neither disagree nor agree” have the same number and is in the middle of the scale from “fully disagree” to “fully agree”

--------------------------------------------------------------------------------------------------------------------------------------------------------------------------------

Type: Numeric (long)

Label: MCTQ14n_n

Range: [1,5] Units: 1

Unique values: 5 Missing .: 4/908

Tabulation: Freq. Numeric Label

54 1 Fully disagree

67 2 Somewhat disagree

164 3 Neither disagree nor agree

239 4 Somewhat agree

380 5 Fully agree

4 . Missing value

--------------------------------------------------------------------------------------------------------------------------------------------------------------------------------

MCTQ_modelling

Calculated MCTQ modelling score: Mean of MCTQ1n_n, MCTQ2n_n and MCTQ3n_n

--------------------------------------------------------------------------------------------------------------------------------------------------------------------------------

Type: Numeric (float)

Range: [1,5] Units: 1.000e-07

Unique values: 13 Missing .: 8/908

Mean: 3.54815

Std. dev.: 1.05164

Percentiles: 10% 25% 50% 75% 90%

2 3 3.66667 4.33333 5

--------------------------------------------------------------------------------------------------------------------------------------------------------------------------------

MCTQ_coaching

Calculated MCTQ coaching score: Mean of MCTQ4n_n, MCTQ5n_n and MCTQ6n_n

--------------------------------------------------------------------------------------------------------------------------------------------------------------------------------

Type: Numeric (float)

Range: [1,5] Units: 1.000e-07

Unique values: 13 Missing .: 10/908

Mean: 3.72049

Std. dev.: 1.01674

Percentiles: 10% 25% 50% 75% 90%

2.33333 3 4 4.66667 5

--------------------------------------------------------------------------------------------------------------------------------------------------------------------------------

MCTQ_articulation

Calculated MCTQ articulation score: Mean of MCTQ7n_n, MCTQ8n_n, and MCTQ9n_n

--------------------------------------------------------------------------------------------------------------------------------------------------------------------------------

Type: Numeric (float)

Range: [1,5] Units: 1.000e-07

Unique values: 13 Missing .: 7/908

Mean: 3.46282

Std. dev.: .975758

Percentiles: 10% 25% 50% 75% 90%

2 3 3.66667 4.33333 4.66667

--------------------------------------------------------------------------------------------------------------------------------------------------------------------------------

MCTQ_exploration

Calculated MCTQ exploration score: Mean of MCTQ10n_n, and MCTQ11n_n

--------------------------------------------------------------------------------------------------------------------------------------------------------------------------------

Type: Numeric (float)

Range: [1,5] Units: .1

Unique values: 9 Missing .: 9/908

Tabulation: Freq. Value

160 1

29 1.5

155 2

73 2.5

186 3

94 3.5

103 4

29 4.5

70 5

9 .

--------------------------------------------------------------------------------------------------------------------------------------------------------------------------------

MCTQ_LE

Calculated MCTQ learning environment score: Mean of MCTQ112n_n, MCTQ13n_n and MCTQ14n_n

--------------------------------------------------------------------------------------------------------------------------------------------------------------------------------

Type: Numeric (float)

Range: [1,5] Units: 1.000e-07

Unique values: 13 Missing .: 5/908

Mean: 3.72499

Std. dev.: 1.10701

Percentiles: 10% 25% 50% 75% 90%

2 3 4 4.66667 5

--------------------------------------------------------------------------------------------------------------------------------------------------------------------------------

MCTQ_tot

Calculated MCTQ total score: Mean of MCTQ1n_n, MCTQ2n_n, MCTQ3n_n, MCTQ4n_n, MCTQ5n_n, MCTQ6n_n, MCTQ7n_n, MCTQ8n_n, MCTQ9n_n, MCTQ10n_n, MCTQ11n_n, MCTQ12n_n, MCTQ13n_n, and MCTQ14n_n

--------------------------------------------------------------------------------------------------------------------------------------------------------------------------------

Type: Numeric (float)

Range: [1,5] Units: 1.000e-07

Unique values: 57 Missing .: 20/908

Mean: 3.48633

Std. dev.: .872868

Percentiles: 10% 25% 50% 75% 90%

2.21429 3 3.57143 4.14286 4.57143

# Overall perception of the supervision encounter

## Raw data

--------------------------------------------------------------------------------------------------------------------------------------------------------------------------------

BestOfMyAbility

Response to: How much do you agree with the following statements: I was allowed to participate to the best of my ability

--------------------------------------------------------------------------------------------------------------------------------------------------------------------------------

Type: String (str26)

Unique values: 6 Missing "": 5/908

Tabulation: Freq. Value

5 ""

323 "Fully agree"

37 "Fully disagree"

21 "Neither agree nor disagree"

101 "Neither disagree nor agree"

307 "Somewhat agree"

114 "Somewhat disagree"

--------------------------------------------------------------------------------------------------------------------------------------------------------------------------------

Trygg

Response to: How much do you agree with the following statements: I felt safe and comfortable during supervision

--------------------------------------------------------------------------------------------------------------------------------------------------------------------------------

Type: String (str26)

Unique values: 6 Missing "": 7/908

Tabulation: Freq. Value

7 ""

368 "Fully agree"

23 "Fully disagree"

23 "Neither agree nor disagree"

105 "Neither disagree nor agree"

291 "Somewhat agree"

91 "Somewhat disagree"

--------------------------------------------------------------------------------------------------------------------------------------------------------------------------------

GoodSupervisor

Response to: How much do you agree with the following statements: I wish we had more supervisors like this one

--------------------------------------------------------------------------------------------------------------------------------------------------------------------------------

Type: String (str26)

Unique values: 6 Missing "": 6/908

Tabulation: Freq. Value

6 ""

354 "Fully agree"

56 "Fully disagree"

54 "Neither agree nor disagree"

145 "Neither disagree nor agree"

215 "Somewhat agree"

78 "Somewhat disagree"

--------------------------------------------------------------------------------------------------------------------------------------------------------------------------------

Learning1

Response to: How was the learning outcome compared to other supervision sessions?

--------------------------------------------------------------------------------------------------------------------------------------------------------------------------------

Type: String (str9)

Unique values: 5 Missing "": 7/908

Tabulation: Freq. Value

7 ""

154 "Excellent"

138 "Fair"

306 "Good"

70 "Poor"

233 "Very good"

## Transformed data

--------------------------------------------------------------------------------------------------------------------------------------------------------------------------------

BestOfMyAbilityn_n

RECODE of BestOfMyAbility_n

Response to: “How much do you agree with the following statements: I was allowed to participate to the best of my ability” with addition of values from disagreeing the most (1) to agreeing the most (5), combining “Neither disagree or agree” and “Neither disagree nor agree” (of which one was used in the survey to Norwegian medical students abroad and the other in the other survey).

--------------------------------------------------------------------------------------------------------------------------------------------------------------------------------

Type: Numeric (long)

Label: BestOfMyAbilityn_n

Range: [1,5] Units: 1

Unique values: 5 Missing .: 5/908

Tabulation: Freq. Numeric Label

37 1 Fully disagree

114 2 Somewhat disagree

122 3 Neither disagree nor agree

307 4 Somewhat agree

323 5 Fully agree

5 . Missing value

--------------------------------------------------------------------------------------------------------------------------------------------------------------------------------

Tryggn_n

RECODE of Trygg_n

Response to: “How much do you agree with the following statements: I felt safe and comfortable during supervision” with addition of values from disagreeing the most (1) to agreeing the most (5), combining “Neither disagree or agree” and “Neither disagree nor agree” (of which one was used in the survey to Norwegian medical students abroad and the other in the other survey).

--------------------------------------------------------------------------------------------------------------------------------------------------------------------------------

Type: Numeric (long)

Label: Tryggn_n

Range: [1,5] Units: 1

Unique values: 5 Missing .: 7/908

Tabulation: Freq. Numeric Label

23 1 Fully disagree

91 2 Somewhat disagree

128 3 Neither disagree nor agree

291 4 Somewhat agree

368 5 Fully agree

7 .

--------------------------------------------------------------------------------------------------------------------------------------------------------------------------------

GoodSupervisorn_n

RECODE of GoodSupervisor_n

Response to: “How much do you agree with the following statements: I wish we had more supervisors like this one” with addition of values from disagreeing the most (1) to agreeing the most (5), combining “Neither disagree or agree” and “Neither disagree nor agree” (of which one was used in the survey to Norwegian medical students abroad and the other in the other survey).

--------------------------------------------------------------------------------------------------------------------------------------------------------------------------------

Type: Numeric (long)

Label: GoodSupervisorn_n

Range: [1,5] Units: 1

Unique values: 5 Missing .: 6/908

Tabulation: Freq. Numeric Label

56 1 Fully disagree

78 2 Somewhat disagree

199 3 Neither disagree nor agree

215 4 Somewhat agree

354 5 Fully agree

6 .

--------------------------------------------------------------------------------------------------------------------------------------------------------------------------------

Learning1_n

Recode of Learning1 (response to: How was the learning outcome compared to other supervision sessions?), adding numerical values from most negative (1) to most positive (5) response

--------------------------------------------------------------------------------------------------------------------------------------------------------------------------------

Type: Numeric (long)

Label: learning_o

Range: [1,5] Units: 1

Unique values: 5 Missing .: 7/908

Tabulation: Freq. Numeric Label

70 1 Poor

138 2 Fair

306 3 Good

233 4 Very good

154 5 Excellent

7 .

# Psychological safety

## Raw data

--------------------------------------------------------------------------------------------------------------------------------------------------------------------------------

PsychSafe1

Response to: How accurate would you say the following statements are: If someone made a mistake it would have been held against them

--------------------------------------------------------------------------------------------------------------------------------------------------------------------------------

Type: String (str31)

Unique values: 7 Missing "": 10/908

Tabulation: Freq. Value

10 ""

51 "Accurate"

275 "Inaccurate"

112 "Moderately accurate"

117 "Moderately inaccurate"

132 "Neither inaccurate nor accurate"

24 "Very accurate"

187 "Very inaccurate"

--------------------------------------------------------------------------------------------------------------------------------------------------------------------------------

PsychSafe2

Response to: How accurate would you say the following statements are: Those present were able to bring up problems and tough issues

--------------------------------------------------------------------------------------------------------------------------------------------------------------------------------

Type: String (str31)

Unique values: 7 Missing "": 11/908

Tabulation: Freq. Value

11 ""

282 "Accurate"

53 "Inaccurate"

198 "Moderately accurate"

58 "Moderately inaccurate"

176 "Neither inaccurate nor accurate"

113 "Very accurate"

17 "Very inaccurate"

--------------------------------------------------------------------------------------------------------------------------------------------------------------------------------

PsychSafe3

Response to: How accurate would you say the following statements are: Those present sometimes reject others for being different

--------------------------------------------------------------------------------------------------------------------------------------------------------------------------------

Type: String (str31)

Unique values: 7 Missing "": 14/908

Tabulation: Freq. Value

14 ""

30 "Accurate"

260 "Inaccurate"

76 "Moderately accurate"

84 "Moderately inaccurate"

174 "Neither inaccurate nor accurate"

8 "Very accurate"

262 "Very inaccurate"

--------------------------------------------------------------------------------------------------------------------------------------------------------------------------------

PsychSafe4

Response to: How accurate would you say the following statements are: It was safe to take a risk

--------------------------------------------------------------------------------------------------------------------------------------------------------------------------------

Type: String (str31)

Unique values: 7 Missing "": 13/908

Tabulation: Freq. Value

13 ""

169 "Accurate"

85 "Inaccurate"

160 "Moderately accurate"

98 "Moderately inaccurate"

305 "Neither inaccurate nor accurate"

45 "Very accurate"

33 "Very inaccurate"

--------------------------------------------------------------------------------------------------------------------------------------------------------------------------------

PsychSafe5

Response to: How accurate would you say the following statements are: It was difficult to ask those present for help

--------------------------------------------------------------------------------------------------------------------------------------------------------------------------------

Type: String (str31)

Unique values: 7 Missing "": 11/908

Tabulation: Freq. Value

11 ""

58 "Accurate"

300 "Inaccurate"

87 "Moderately accurate"

133 "Moderately inaccurate"

92 "Neither inaccurate nor accurate"

22 "Very accurate"

205 "Very inaccurate"

--------------------------------------------------------------------------------------------------------------------------------------------------------------------------------

PsychSafe6

Response to: How accurate would you say the following statements are: No one present deliberately acted in a way that undermined my efforts

--------------------------------------------------------------------------------------------------------------------------------------------------------------------------------

Type: String (str31)

Unique values: 7 Missing "": 15/908

Tabulation: Freq. Value

15 ""

285 "Accurate"

47 "Inaccurate"

90 "Moderately accurate"

43 "Moderately inaccurate"

163 "Neither inaccurate nor accurate"

228 "Very accurate"

37 "Very inaccurate"

--------------------------------------------------------------------------------------------------------------------------------------------------------------------------------

PsychSafe7

Response to: How accurate would you say the following statements are: My unique skills and talents were valued and utilised by those present

--------------------------------------------------------------------------------------------------------------------------------------------------------------------------------

Type: String (str31)

Unique values: 7 Missing "": 13/908

Tabulation: Freq. Value

13 ""

182 "Accurate"

83 "Inaccurate"

169 "Moderately accurate"

84 "Moderately inaccurate"

285 "Neither inaccurate nor accurate"

54 "Very accurate"

38 "Very inaccurate"

## Transformed data

--------------------------------------------------------------------------------------------------------------------------------------------------------------------------------

PsychSafe1_n

Recode of PsychSafe1 (Response to: How accurate would you say the following statements are: If someone made a mistake it would have been held against them) so that numeric value is added from most negative (1) to most positive (7) response

--------------------------------------------------------------------------------------------------------------------------------------------------------------------------------

Type: Numeric (long)

Label: PsychSafety_or

Range: [1,7] Units: 1

Unique values: 7 Missing .: 10/908

Tabulation: Freq. Numeric Label

24 1 Very accurate

51 2 Accurate

112 3 Moderately accurate

132 4 Neither inaccurate nor accurate

117 5 Moderately inaccurate

275 6 Inaccurate

187 7 Very inaccurate

10 . Missing value

--------------------------------------------------------------------------------------------------------------------------------------------------------------------------------

PsychSafe2_n

Recode of PsychSafe2 (Response to: How accurate would you say the following statements are: Those present were able to bring up problems and tough issues) so that numeric value is added from most negative (1) to most positive (7) response

--------------------------------------------------------------------------------------------------------------------------------------------------------------------------------

Type: Numeric (long)

Label: PsychSafety_o

Range: [1,7] Units: 1

Unique values: 7 Missing .: 11/908

Tabulation: Freq. Numeric Label

17 1 Very inaccurate

53 2 Inaccurate

58 3 Moderately inaccurate

176 4 Neither inaccurate nor accurate

198 5 Moderately accurate

282 6 Accurate

113 7 Very accurate

11 . Missing value

--------------------------------------------------------------------------------------------------------------------------------------------------------------------------------

PsychSafe3_n

Recode of PsychSafe3 (Response to: How accurate would you say the following statements are: Those present sometimes reject others for being different) so that numeric value is added from most negative (1) to most positive (7) response

--------------------------------------------------------------------------------------------------------------------------------------------------------------------------------

Type: Numeric (long)

Label: PsychSafety_or

Range: [1,7] Units: 1

Unique values: 7 Missing .: 14/908

Tabulation: Freq. Numeric Label

8 1 Very accurate

30 2 Accurate

76 3 Moderately accurate

174 4 Neither inaccurate nor accurate

84 5 Moderately inaccurate

260 6 Inaccurate

262 7 Very inaccurate

14 . Missing value

--------------------------------------------------------------------------------------------------------------------------------------------------------------------------------

PsychSafe4_n

Recode of PsychSafe4 (Response to: How accurate would you say the following statements are: It was safe to take a risk) so that numeric value is added from most negative (1) to most positive (7) response

--------------------------------------------------------------------------------------------------------------------------------------------------------------------------------

Type: Numeric (long)

Label: PsychSafety_o

Range: [1,7] Units: 1

Unique values: 7 Missing .: 13/908

Tabulation: Freq. Numeric Label

33 1 Very inaccurate

85 2 Inaccurate

98 3 Moderately inaccurate

305 4 Neither inaccurate nor accurate

160 5 Moderately accurate

169 6 Accurate

45 7 Very accurate

13 . Missing value

--------------------------------------------------------------------------------------------------------------------------------------------------------------------------------

PsychSafe5_n

Recode of PsychSafe5 (Response to: How accurate would you say the following statements are: It was difficult to ask those present for help) so that numeric value is added from most negative (1) to most positive (7) response

--------------------------------------------------------------------------------------------------------------------------------------------------------------------------------

Type: Numeric (long)

Label: PsychSafety_or

Range: [1,7] Units: 1

Unique values: 7 Missing .: 11/908

Tabulation: Freq. Numeric Label

22 1 Very accurate

58 2 Accurate

87 3 Moderately accurate

92 4 Neither inaccurate nor accurate

133 5 Moderately inaccurate

300 6 Inaccurate

205 7 Very inaccurate

11 . Missing value

--------------------------------------------------------------------------------------------------------------------------------------------------------------------------------

PsychSafe6_n

Recode of PsychSafe1 (Response to: How accurate would you say the following statements are: No one present deliberately acted in a way that undermined my efforts) so that numeric value is added from most negative (1) to most positive (7) response

--------------------------------------------------------------------------------------------------------------------------------------------------------------------------------

Type: Numeric (long)

Label: PsychSafety_o

Range: [1,7] Units: 1

Unique values: 7 Missing .: 15/908

Tabulation: Freq. Numeric Label

37 1 Very inaccurate

47 2 Inaccurate

43 3 Moderately inaccurate

163 4 Neither inaccurate nor accurate

90 5 Moderately accurate

285 6 Accurate

228 7 Very accurate

15 . Missing value

--------------------------------------------------------------------------------------------------------------------------------------------------------------------------------

PsychSafe7_n

Recode of PsychSafe1 (Response to: How accurate would you say the following statements are: My unique skills and talents were valued and utilised by those present) so that numeric value is added from most negative (1) to most positive (7) response

--------------------------------------------------------------------------------------------------------------------------------------------------------------------------------

Type: Numeric (long)

Label: PsychSafety_o

Range: [1,7] Units: 1

Unique values: 7 Missing .: 13/908

Tabulation: Freq. Numeric Label

38 1 Very inaccurate

83 2 Inaccurate

84 3 Moderately inaccurate

285 4 Neither inaccurate nor accurate

169 5 Moderately accurate

182 6 Accurate

54 7 Very accurate

13 . Missing value

--------------------------------------------------------------------------------------------------------------------------------------------------------------------------------

PS_Score

Calculated psychological safety score: Mean of PsychSafe1_n, PsychSafe2_n, PsychSafe3_n, PsychSafe4_n, PsychSafe5_n, PsychSafe6_n and PsychSafe7_n

--------------------------------------------------------------------------------------------------------------------------------------------------------------------------------

Type: Numeric (float)

Range: [1.4285715,7] Units: 1.000e-07

Unique values: 38 Missing .: 22/908

Mean: 4.93373

Std. dev.: .954618

Percentiles: 10% 25% 50% 75% 90%

3.71429 4.28571 5 5.57143 6.14286

# Supervisor characteristic and supervision context

## Raw data

--------------------------------------------------------------------------------------------------------------------------------------------------------------------------------

Whatprofessiondidthesupervis

Response to: What profession did the supervisor have?

--------------------------------------------------------------------------------------------------------------------------------------------------------------------------------

Type: String (str30)

Unique values: 8 Missing "": 5/908

Tabulation: Freq. Value

5 ""

1 "Academic"

1 "Assistant"

4 "Do not remember/do not know"

845 "Doctor"

41 "Medical student"

6 "Nurse"

4 "Other health care professional"

1 "Other, please specify"

--------------------------------------------------------------------------------------------------------------------------------------------------------------------------------

BK

Free text response to specify supervisor profession

--------------------------------------------------------------------------------------------------------------------------------------------------------------------------------

Type: String (str22)

Unique values: 8 Missing "": 899/908

Tabulation: Freq. Value

899 ""

1 "ASO"

1 "Assistant"

1 "Clinical chief"

1 "Interne"

1 "LIS1"

1 "Part of PhD-programme."

1 "Professor, doctor"

2 "Resident"

--------------------------------------------------------------------------------------------------------------------------------------------------------------------------------

Whatwasthedoctor39slevel

Response to: What was the doctor’s level of experience?

--------------------------------------------------------------------------------------------------------------------------------------------------------------------------------

Type: String (str11)

Unique values: 5 Missing "": 70/908

Tabulation: Freq. Value

70 ""

113 "2-4 years"

193 "5-10 years"

42 "<2 years"

387 ">10 years"

103 "Do not know"

--------------------------------------------------------------------------------------------------------------------------------------------------------------------------------

Isthesupervisoremployedbyth

Response to: Is the supervisor employed by the university?

--------------------------------------------------------------------------------------------------------------------------------------------------------------------------------

Type: String (str11)

Unique values: 3 Missing "": 7/908

Tabulation: Freq. Value

7 ""

133 "Do not know"

220 "No"

548 "Yes"

--------------------------------------------------------------------------------------------------------------------------------------------------------------------------------

Whatwasthesupervisor39sge

Response to: What was the supervisor’s gender?

--------------------------------------------------------------------------------------------------------------------------------------------------------------------------------

Type: String (str15)

Unique values: 5 Missing "": 5/908

Tabulation: Freq. Value

5 ""

2 "Do not know"

12 "Do not remember"

394 "Female"

493 "Male"

2 "Non-binary"

--------------------------------------------------------------------------------------------------------------------------------------------------------------------------------

NoPrevSupervision

Response to: On how many previous occasions had the supervisor supervised you?

--------------------------------------------------------------------------------------------------------------------------------------------------------------------------------

Type: String (str22)

Unique values: 6 Missing "": 5/908

Tabulation: Freq. Value

5 ""

332 "0"

193 "1-2"

154 "3-5"

83 "5-10"

94 ">10"

47 "Unsure/do not remember"

--------------------------------------------------------------------------------------------------------------------------------------------------------------------------------

Inwhichsubjectareadidthe

Response to: In which subject (area) did the supervision take place?

--------------------------------------------------------------------------------------------------------------------------------------------------------------------------------

Type: String (str26)

Unique values: 17 Missing "": 14/908

Set response with the following options:

Anaesthesia

Dermatology

Ear, nose and throat (ENT)

Family medicine

Internal medicine

Neurology

Obstetrics and gynaecology

Oncology

Ophthalmology

Orthopaedics

Palliative care

Paediatrics

Psychiatry

Radiology

Rehabilitation

Surgery

Other

## Transformed data

--------------------------------------------------------------------------------------------------------------------------------------------------------------------------------

SupervisorProfession

Response to question “What profession did the supervisor have?” with addition of numeric value

--------------------------------------------------------------------------------------------------------------------------------------------------------------------------------

Type: Numeric (long)

Label: SupervisorProfession

Range: [1,8] Units: 1

Unique values: 8 Missing .: 5/908

Tabulation: Freq. Numeric Label

1 1 Academic

1 2 Assistant

4 3 Do not remember/do not know

845 4 Doctor

41 5 Medical student

6 6 Nurse

4 7 Other health care professional

1 8 Other, please specify

5 . Missing value

--------------------------------------------------------------------------------------------------------------------------------------------------------------------------------

SupervisorProfessionRank

RECODE of SupervisorProfession (Response to question “What profession did the supervisor have?”) so that lowest value to the least education and highest value to most education

--------------------------------------------------------------------------------------------------------------------------------------------------------------------------------

Type: Numeric (long)

Label: SupervisorProfessionRank, but 1 nonmissing value is not labeled

Range: [0,4] Units: 1

Unique values: 5 Missing .: 12/908

Tabulation: Freq. Numeric Label

107 0 do not know

12 1 Other

197 2 intern/resident

193 3 doctor

387 4

12 . Missing value

.

--------------------------------------------------------------------------------------------------------------------------------------------------------------------------------

DoctorSeniority

Response to question “What was the doctor’s level of experience?” with addition of numeric value

--------------------------------------------------------------------------------------------------------------------------------------------------------------------------------

Type: Numeric (long)

Label: DoctorSeniority

Range: [1,5] Units: 1

Unique values: 5 Missing .: 70/908

Tabulation: Freq. Numeric Label

113 1 2-4 years

193 2 5-10 years

42 3 <2 years

387 4 >10 years

103 5 Do not know

70 . Missing value

--------------------------------------------------------------------------------------------------------------------------------------------------------------------------------

DoctorSeniorityRank

RECODE of DoctorSeniority (Response to question: “What was the doctor’s level of experience?”) so that numeric value goes from lowest for shortest experience to highest for longest experience

--------------------------------------------------------------------------------------------------------------------------------------------------------------------------------

Type: Numeric (long)

Label: DoctorSeniorityRank

Range: [0,4] Units: 1

Unique values: 4 Missing .: 70/908

Tabulation: Freq. Numeric Label

103 0 do not know

155 2 up to 4 year experience

193 3 5-10 years experience

387 4 >10 years experience

70 .

--------------------------------------------------------------------------------------------------------------------------------------------------------------------------------

SupervisorSeniority

Combination of SupervisorProfession and SupervisorSeniority, so that those who responded “doctor” to supervisor profession are group according to the years of experience they responded the supervisor had

--------------------------------------------------------------------------------------------------------------------------------------------------------------------------------

Type: Numeric (long)

Label: SupervisorSeniority

Range: [0,4] Units: 1

Unique values: 5 Missing .: 12/908

Tabulation: Freq. Numeric Label

387 0 >10 years’ experience

193 1 doctor 5-10 years’ experience

197 2 Intern/resident/doctor <5 years’ experience

12 3 Other

107 4 Do not know

12 . Missing value

--------------------------------------------------------------------------------------------------------------------------------------------------------------------------------

UniEmployee

Response to question “Is the supervisor employed by the university?” with addition of numeric value

--------------------------------------------------------------------------------------------------------------------------------------------------------------------------------

Type: Numeric (long)

Label: UniEmployee

Range: [1,3] Units: 1

Unique values: 3 Missing .: 7/908

Tabulation: Freq. Numeric Label

133 1 Do not know

220 2 No

548 3 Yes

7 . Missing value

--------------------------------------------------------------------------------------------------------------------------------------------------------------------------------

SupervisorGender

Response to question “What was the supervisor’s gender?” with addition of numeric value

--------------------------------------------------------------------------------------------------------------------------------------------------------------------------------

Type: Numeric (long)

Label: SupervisorGender

Range: [1,5] Units: 1

Unique values: 5 Missing .: 5/908

Tabulation: Freq. Numeric Label

2 1 Do not know

12 2 Do not remember

394 3 Female

493 4 Male

2 5 Non-binary

5 . Missing value

--------------------------------------------------------------------------------------------------------------------------------------------------------------------------------

PreviousSupervision

Response to question: “On how many previous occasions had the supervisor supervised you?” with addition of numeric value for each category

--------------------------------------------------------------------------------------------------------------------------------------------------------------------------------

Type: Numeric (long)

Label: DummyDD

Range: [1001,1006] Units: 1

Unique values: 6 Missing .: 5/908

Tabulation: Freq. Numeric Label

332 1001 0

193 1002 1-2

154 1003 3-5

83 1004 5-10

94 1005 >10

47 1006 Unsure/do not remember

5 . Missing value

--------------------------------------------------------------------------------------------------------------------------------------------------------------------------------

PreviousSupervision_g

RECODE of PreviousSupervision

Response to question: “On how many previous occasions had the supervisor supervised you?” grouped into 4 groups

--------------------------------------------------------------------------------------------------------------------------------------------------------------------------------

Type: Numeric (long)

Label: PreviousSupervision_g, but 1 nonmissing value is not labeled

Range: [0,3] Units: 1

Unique values: 4 Missing .: 52/908

Tabulation: Freq. Numeric Label

332 0 0

347 1 1-5

83 2 5-10

94 3 >10

52 . Missing value

--------------------------------------------------------------------------------------------------------------------------------------------------------------------------------

Speciality

Response to question “In which subject (area) did the supervision take place?” with addition of numeric value

--------------------------------------------------------------------------------------------------------------------------------------------------------------------------------

Type: Numeric (long)

Label: Speciality

Range: [1,17] Units: 1

Unique values: 17 Missing .: 14/908

Numeric Label

1 Anaesthesia

2 Dermatology

3 Ear, nose and throat (ENT)

4 Family medicine

5 Internal medicine

6 Neurology

7 Obstetrics and gynaecology

8 Oncology

9 Ophthalmology

10 Orthopaedics

11 Other

12 Palliative care

13 Paediatrics

14 Psychiatry

15 Radiology

16 Rehabilitation

17 Surgery

--------------------------------------------------------------------------------------------------------------------------------------------------------------------------------

Speciality40

RECODE of Speciality

Response to question: “In which subject (area) did the supervision take place?”, but all response options with less than 40 responses included in the “other” category.

--------------------------------------------------------------------------------------------------------------------------------------------------------------------------------

Type: Numeric (long)

Label: Speciality40

Range: [0,17] Units: 1

Unique values: 8 Missing .: 14/908

Tabulation: Freq. Numeric Label

249 0 Other

74 4 Family Medicine

246 5 Internal Medicine

72 6 Neurology

44 7 Obstetrics and gynaecology

43 10 Orthopaedics

92 13 Paediatrics

74 17 Surgery

14 . Missing value

.
