# Supplementary material for: Psychological safety in European medical students’ last supervised patient encounter: A cross-sectional survey
Source: PLoS One. 2023 Apr 27;18(4):e0285014. doi: 10.1371/journal.pone.0285014 (PMC10138856; doi:10.1371/journal.pone.0285014)
Supplement: Attachment — Submitted filename: Response to Reviewers.docx [file pone.0285014.s003.docx]

Journal requirements:

When submitting your revision, we need you to address these additional requirements.

1.  Please ensure that your manuscript meets PLOS ONE's style requirements, including those for file naming. The PLOS ONE style templates can be found at

<https://journals.plos.org/plosone/s/file?id=wjVg/PLOSOne_formatting_sample_main_body.pdf>  and

<https://journals.plos.org/plosone/s/file?id=ba62/PLOSOne_formatting_sample_title_authors_affiliations.pdf>

2. Please provide additional details regarding participant consent. In the ethics statement in the Methods and online submission information, please ensure that you have specified (1) whether consent was informed and (2) what type you obtained (for instance, written or verbal, and if verbal, how it was documented and witnessed). If your study included minors, state whether you obtained consent from parents or guardians. If the need for consent was waived by the ethics committee, please include this information.

In the “design and ethics” section we have written that (1) participants received written information about the study, i.e. had information about the study before providing consent, and (2) that they had to click “send” to submit their responses and they were informed that clicking “send” was considered consent, i.e. they gave a digital consent without a signature (to ensure anonymity). We would be happy to rewrite this section so that it fits your requirements but are unsure what needs to be changed.

If you are reporting a retrospective study of medical records or archived samples, please ensure that you have discussed whether all data were fully anonymized before you accessed them and/or whether the IRB or ethics committee waived the requirement for informed consent. If patients provided informed written consent to have data from their medical records used in research, please include this information.

3. In your Data Availability statement, you have not specified where the minimal data set underlying the results described in your manuscript can be found. PLOS defines a study's minimal data set as the underlying data used to reach the conclusions drawn in the manuscript and any additional data required to replicate the reported study findings in their entirety. All PLOS journals require that the minimal data set be made fully available. For more information about our data policy, please see <http://journals.plos.org/plosone/s/data-availability>.

Upon re-submitting your revised manuscript, please upload your study’s minimal underlying data set as either Supporting Information files or to a stable, public repository and include the relevant URLs, DOIs, or accession numbers within your revised cover letter. For a list of acceptable repositories, please see [http://journals.plos.org/plosone/s/data-availability#loc-recommended-repositories](http://journals.plos.org/plosone/s/data-availability#loc-recommended-repositories). Any potentially identifying patient information must be fully anonymized.

Important: If there are ethical or legal restrictions to sharing your data publicly, please explain these restrictions in detail. Please see our guidelines for more information on what we consider unacceptable restrictions to publicly sharing data: [http://journals.plos.org/plosone/s/data-availability#loc-unacceptable-data-access-restrictions](http://journals.plos.org/plosone/s/data-availability#loc-unacceptable-data-access-restrictions). Note that it is not acceptable for the authors to be the sole named individuals responsible for ensuring data access.

We will update your Data Availability statement to reflect the information you provide in your cover letter.

We have attached the data files (“Dataset” and “Data codebook”) to our upload.

[Note: HTML markup is below. Please do not edit.]

Reviewers' comments:

Reviewer's Responses to Questions

**Comments to the Author**

1. Is the manuscript technically sound, and do the data support the conclusions?

The manuscript must describe a technically sound piece of scientific research with data that supports the conclusions. Experiments must have been conducted rigorously, with appropriate controls, replication, and sample sizes. The conclusions must be drawn appropriately based on the data presented.

Reviewer #1: Yes

Reviewer #2: Partly

2. Has the statistical analysis been performed appropriately and rigorously?

Reviewer #1: Yes

Reviewer #2: Yes

3. Have the authors made all data underlying the findings in their manuscript fully available?

The [PLOS Data policy](http://www.plosone.org/static/policies.action#sharing) requires authors to make all data underlying the findings described in their manuscript fully available without restriction, with rare exception (please refer to the Data Availability Statement in the manuscript PDF file). The data should be provided as part of the manuscript or its supporting information, or deposited to a public repository. For example, in addition to summary statistics, the data points behind means, medians and variance measures should be available. If there are restrictions on publicly sharing data—e.g. participant privacy or use of data from a third party—those must be specified.

Reviewer #1: Yes

Reviewer #2: Yes

4. Is the manuscript presented in an intelligible fashion and written in standard English?

PLOS ONE does not copyedit accepted manuscripts, so the language in submitted articles must be clear, correct, and unambiguous. Any typographical or grammatical errors should be corrected at revision, so please note any specific errors here.

Reviewer #1: Yes

Reviewer #2: Yes

5. Review Comments to the Author

Thank you to both reviewers for giving useful feedback that helped improve our article.

Please use the space provided to explain your answers to the questions above. You may also include additional comments for the author, including concerns about dual publication, research ethics, or publication ethics. (Please upload your review as an attachment if it exceeds 20,000 characters)

Reviewer #1:

- Are there any impact/issues on psychological safety among medical students? If yes, please provide the evidence in order to show that this study is important
  - We have rewritten the introduction to make clearer what research has been done in the past and why this study is important. In particular, we have now stated that:

«In medical students’ supervised encounters with patients, where education meets health care, there was an association between the students’ perception of learning and psychological safety [9].

This indicates that promoting psychological safety in medical students’ supervised encounters with patients is important. However, we have found no studies looking at the association between psychological safety and medical students’ experiences with other aspects of supervised patient encounters. Such knowledge can be helpful in unravelling what can be done to promote psychological safety in this setting.»

- . We hope this answers reviewer 1s question.
- Provide some explanation related to your variables.
  - We have added several paragraphs in the methods section to have a more detailed description of which variables were included and what they entail.
- What method that the researcher used in selecting the participants?
  - We have revised the first sentence of the “design and ethics” section so that it now reads “This was a cross-sectional survey where European medical students were invited to complete an online questionnaire.” Together with the “participants, recruitment, and data collection” section, particularly the paragraph “To recruit participants, the online survey was distributed as web-links in e-mails and on social media by student representatives in national medical student associations in the International Federation of Medical Students Associations (IFMSA) between March and October 2020. Individual students were also encouraged to share the link with their peers.” We hope it is now clear that we did not select individual participants but invited all European medical students we could reach.
- State the reliability result for Maastricht Clinical Teaching Questionnaire (MCTQ) in this study
  - In the new paragraphs on variables we have now added that the MCTQ “subscales had Cronbach’s alphas ranging from 0.74 to 0.87 in this study.” We have also added the Cronbach alpha value for the psychological safety scale.
- Under Questionnaire and variables, you mentioned that there are 12 independent variables were used in this study. Please what are the 12 independent variables.
  - We have added several paragraphs in the methods section to have a more detailed description of which variables were included and what they entail.
- For the respondents, ‘not male’ refer to female or what? Please explain it
  - We have added the sentences “In post-graduate medical education, research suggest gender impacts trainees' perception of psychological safety [15]. Male was chosen as the comparator, as some studies suggest males feel more psychologically safe [15].” We hope this explains our choice and what it entails.

Reviewer #2: The authors investigated the relationship between supervisors’ attributes and behaviors and students’ psychological safety in clinical educational settings, an area of interest to readers. A sufficient number of data have been analyzed robustly quantitatively, and the methods are clear.
However, I have concerns regarding the description of the purpose of the study.
The authors cited literature and stated that in nonmedical education, studies showed that psychological safety influences students’ learning behaviors and performance, whereas, in clinical medical education, only a few studies demonstrated the effects of psychological safety on education. Readers of the Introduction section may assume that the purpose of the current study is “to determine how psychological safety affects education in the medical education area.”

However, the authors investigated factors influencing psychological safety in the clinical education field, rather than the impact of psychological safety on education.

1 Therefore, first, please clarify whether the purpose of this study is to identify factors influencing psychological safety in the clinical education field or to identify the impact of psychological safety on clinical education for medical students.
2 If the latter, consider whether the method is appropriate to achieve the purpose.
3 If it is the former, I would like you to describe a logical and scientific reason why the authors investigated the factors affecting psychological safety in clinical education, especially regarding supervision. For example, how about citing previous studies on the effects of supervision on psychological safety outside clinical education settings?

The reviewers’ comments made us realise that our introduction was not up to the wanted standard, lacking a clear explanation of why this study is relevant and novel, as well as exactly what was investigated. We hope our revisions has made this clear as we now argue why psychological safety is important to medical students’ learning in supervised patient encounters and we need to learn more about how psychological safety comes to exist in this context.

6. PLOS authors have the option to publish the peer review history of their article ([what does this mean?](https://journals.plos.org/plosone/s/editorial-and-peer-review-process#loc-peer-review-history)). If published, this will include your full peer review and any attached files.


If you choose “no”, your identity will remain anonymous but your review may still be made public.


**Do you want your identity to be public for this peer review?** For information about this choice, including consent withdrawal, please see our [Privacy Policy](https://www.plos.org/privacy-policy).

Reviewer #1: No

Reviewer #2: No
